# Development of Collagen-Based 3D Matrix for Gastrointestinal Tract-Derived Organoid Culture

**DOI:** 10.1155/2019/8472712

**Published:** 2019-06-13

**Authors:** Joo Hyun Jee, Dong Hyeon Lee, Jisu Ko, Soojung Hahn, Sang Yun Jeong, Han Kyung Kim, Enoch Park, Seon Young Choi, Sukin Jeong, Joong Woon Lee, Han-Jun Cho, Min-Soo Kwon, Jongman Yoo

**Affiliations:** ^1^Department of Microbiology, School of Medicine, CHA University, Seongnam-si, Gyeonggi-do 13488, Republic of Korea; ^2^CHA Organoid Research Center, School of Medicine, CHA University, Seongnam-si, Gyeonggi-do 13488, Republic of Korea; ^3^Department of Physiology, School of Medicine, CHA University, Seongnam-si, Gyeonggi-do 13488, Republic of Korea; ^4^Department of Pharmacology, School of Medicine, CHA University, Seongnam-si, Gyeonggi-do 13488, Republic of Korea

## Abstract

Organoid is a cell organization grown in a three-dimensional (3D) culture system which represents all characteristics of its origin. However, this organ-like structure requires supporting matrix to maintain its characteristics and functions. Matrigel, derived from mouse sarcoma, has often been used as the supporting matrix for organoids, but the result may not be desirable for clinical applications because of the unidentified components from the mouse sarcoma. On the other hand, natural characteristics of collagen emphasize toxic-free friendly niche to both organoid and normal tissue. Hence, this study attempts to develop a new, collagen-based matrix that may substitute Matrigel in organoid culture. Collagen-based matrix was made, using type 1 collagen, Ham's F12 nutrient mixture, and bicarbonate. Then, characteristics of mouse colon organoids were analyzed by morphology and quantitative messenger RNA (mRNA) expression, revealing that the mouse colon organoids grown in the collagen-based matrix and in Matrigel had quite similar morphology, specific markers, and proliferative rates. Mouse small intestine–derived organoids, stomach-derived organoids, and human colon–derived organoids were also cultured, all of which were successfully grown in the collagen-based matrix and had similar properties compared to those cultured in Matrigel. Furthermore, possibility of organoid transplantation was observed. When mouse colon organoids were transplanted with collagen matrix into the EDTA-colitis mouse model, colon organoids were successfully engrafted in damaged tissue. For that reason, the use of collagen-based matrix in organoid culture will render organoid cultivation less expensive and clinically applicable.

## 1. Introduction

An organoid is a cellular structure, derived from self-organizing stem cells in vitro. It comprises cell populations, three-dimensional (3D) architectures, and organ-specific functions of the originating organs. Advances in 3D culture methodology using epithelial stem cells isolated in the gastrointestinal tract have made the generation of epithelial organoids possible [1, 2]. These organoids reproduce *in vivo* physiology, containing all the tissue-specific cell types derived from epithelial stem cells and their proliferative properties. These models may provide an *in vitro* platform for studying pathophysiology, screening drug efficacy, and testing drug toxicity [3]. Organoids may also be used as therapeutic agents for damaged epithelial tissues, because these recovered intestinal epithelial tissues in the animal models of inflammatory bowel diseases [4–6].

Signaling induced by extracellular matrix (ECM) proteins such as laminin and collagen is vital for stem cells to proliferate, differentiate, and perform the proper functions. Therefore, an ECM must support epithelial stem cells to propagate and maintain its form as an organoid. Currently, the extracellular matrix used for making most organoids is Matrigel, as the original method to derive intestinal epithelial organoids uses Matrigel as a 3D scaffold [7]. Matrigel is a gelatinous protein mixture secreted from mouse EHS sarcoma cells and contains laminin, entactin, collagen, proteoglycan, and various growth factors which may induce extracellular matrix signaling suitable for organoid formation. However, there is diverse evidence that Matrigel-containing materials may promote angiogenesis when injected *in vivo* [8–11]. The ingredients in Matrigel are poorly defined, such that it is difficult to predict its effect on the human body, and the batch-to-batch variation is so severe that it is difficult to plan reproducible studies [12]. Because of these risks, materials using Matrigel cannot be approved by the Food and Drug Administration (FDA), and it is difficult to obtain approval for its use in clinical trials.

Collagen is a universal component of extracellular matrix constituent proteins in living organisms and has various roles such as supporting cell division, migration, and differentiation in the body. Collagen has been used for a variety of cultures, including 2D, 3D, many kinds of cells, and organoids, as well as in medical applications, including extended cultivation of therapeutic cells, artificial skin, cosmetics, and reconstruction [13]. By replacing Matrigel with a collagen-based matrix for organoid culture, it is possible to cultivate organoids at a level suitable for clinical applications. For this reason, this study investigates whether it is possible to expand organoids from the large intestine, small intestine, and stomach in a collagen-based matrix instead of in Matrigel.

## 2. Materials and Methods

### 2.1. Materials

Type I collagen gel (cellMatrix type 1-A) was purchased from Nitta Gelatin, Inc. (Osaka, Japan). Ham's F-12 nutrient mix, advanced DMEM/F12, N-2, B-27, gentamicin/amphotericin B, HEPES, and GlutaMAX-I were obtained from Gibco (Waltham, MA, USA). Matrigel was acquired from BD Biosciences (Franklin Lakes, NJ, USA). mEGF and mNoggin were purchased from PeproTech (Rocky Hill, NJ, USA). CHIR99021, [Leu15]-Gastrin 1, SB202190, nicotinamide, N-acetylcysteine, and thiazovivin were obtained from Sigma-Aldrich (St. Louis, MO, USA). A83-01 was acquired from Tocris Bioscience (Bristol, UK). Y-27632 and Gentle Cell Dissociation Reagent were purchased from STEMCELL Technologies (Vancouver, Canada). Penicillin and streptomycin were acquired from Welgene Inc. (Gyeongsan-si, Republic of Korea). FGF10 was purchased from ATGen (Seongnam, Republic of Korea).

### 2.2. Animals

C57BL/6-Tg(CAG-EGFP) mice [14] and male C57BL/6 mice were obtained from Japan SLC (Shizuoka, Japan) and Orient Bio (Seongnam, Republic of Korea), respectively, and the animals were handled in the CHA University animal facility. This experiment was approved and conducted according to the regulations and guidelines of CHA University Institutional Animal Care and Use Committee.

### 2.3. Preparation of Collagen-Based Matrix for Organoid Culture

The collagen-based matrix was prepared by mixing type I collagen gel (3 mg/mL) from porcine tendon (I) with Ham's F-12 Nutrient Mix (II) and 1 N NaHCO3 (III) in a ratio of 8:1:1, respectively. The mixture was followed in order of (I), (II), and (III) procedure immediately before use.

### 2.4. Isolation and Culture of Mouse Colon Organoids

Mouse colon organoid had been developed previously [15]. Mouse colon crypts were obtained from 5- to 7-week-old C57BL/6 mice. The colon was rinsed with a chelating buffer (2 mM EDTA in DPBS) for 30 minutes, and the crypts were isolated by vigorous inverting for several minutes with a dissociation buffer (1% D-sorbitol and 1% sucrose in DPBS). The isolated crypts were filtered through a 70 *μ*m cell strainer and embedded with Matrigel or a collagen-based matrix at a 1 : 1 ratio, then plated in multiwell plates. After polymerization of the matrices, mouse colon organoid growth medium was added and incubated at 37°C with a 5% CO2 humidified incubator. The culture medium was replaced every 2 days.

Basal growth media supplemented with 10% R-Spondin 1 conditioned medium from HA-R-Spondin 1-Fc 293T cells (Trevigen, Gaithersburg, MD, USA), 50 ng/ml mEGF, 100 ng/ml mNoggin, 50% Wnt-3A conditioned medium from L-Wnt-3A cell (ATCC, Manassas, VA, USA), 500 nM A83-01, 10 *μ*M Y-27632, and 2.5 *μ*M CHIR99021. CHIR99021 was subtracted 2 days after subculture (Table 1).

### 2.5. Isolation and Culture of Mouse Small Intestinal Organoid

Except for the isolation of the crypts, preparation of the small intestine crypts from the mouse small intestine was performed as described above. Small intestine crypts were isolated with Gentle Cell Dissociation Reagent after the tissues were cut longitudinally into 5 mm pieces from the proximal end to the distal end and cut transversely to be opened. Isolated crypts were filtered through a cell strainer before being mixed with Matrigel or a collagen-based matrix at a ratio of 1 : 1 and seeded in multiwell plates. After polymerization of the matrices, mouse small intestinal organoid growth medium was added.

The composition of the mouse small intestinal organoid growth medium is as follows: basal growth media supplemented with 10% R-Spondin 1 conditioned medium, mEGF, mNoggin, A83-01, Y-27632, and CHIR99021 (Table 1).

### 2.6. Isolation and Culture of Mouse Stomach Organoid

Mouse stomach tissue was obtained from the pyloric antrum to the fundus. The tissue was then opened and washed several times in ice-cold DPBS. To isolate the gastric glands, the tissue was put into 5 ml of ice-cold chelating buffer (5 mM EDTA in DPBS) and incubated for 2 hours on an orbital shaker at room temperature. After incubation, 5 ml of dissociation buffer was added and gently inverted by hand for 1–2 minutes. Next, the gastric glands were filtered through a cell strainer and plated with Matrigel or a collagen-based matrix at a ratio of 1 : 1 and seeded in multiwell plates. After polymerization of the matrices, mouse stomach organoid growth medium was added.

The composition of the mouse stomach organoid growth medium was as follows: basal growth media supplemented with 10% R-Spondin 1 conditioned medium, mEGF, mNoggin, 50% Wnt-3A conditioned medium, 500 nM A83-01, 10 *μ*M Y-27632, 10 nM [Leu15]-Gastrin 1, 200 ng/ml FGF10, and 2.5 *μ*M CHIR99021 (Table 1).

### 2.7. Isolation and Culture of Human Colon Organoid

The protocol was conducted in accordance with the Declaration of Helsinki and was approved by the institutional review board at CHA Bundang Hospital. The experiment used 2 × 2 cm^2^ surgical specimens offered by the CHA Bundang Hospital. The specimens, submucosa layers, and muscle parts were carefully removed and washed. The mucosa was then cut into small pieces and washed with DPBS containing gentamicin/amphotericin B. To remove villi and debris, an additional of 3–4 washing steps with a chelating buffer was added. The mucosa was covered with a chelating buffer and incubated at room temperature for 30 minutes, gently inverted on the shaker. To obtain intestinal crypts, mucosa parts were gently scraped with curved forceps. The intestinal crypts were then filtered through 150 *μ*m mesh openings (Dynamic Aqua-Supply, Surrey, Canada) 2 times. After filtration, the supernatant of the crypts was discarded. This was followed by centrifugation at 250 g for 5 minutes at 4°C. Next, the colon crypts were filtered through a cell strainer and plated with Matrigel or a collagen-based matrix at a ratio of 1 : 1 and seeded in multiwell plates. After polymerization of the matrices, a human colon organoid growth medium was added.

The composition of human colon organoid growth medium was as follows: basal growth media with 50% Wnt-3A conditioned medium, 10% R-Spondin 1 conditioned medium, mNoggin 100 ng/ml, mEGF 50 ng/ml, 1% bovine serum albumin, 500 nM A83-01, 10 *μ*M SB202190, 10 nM [Leu15]-Gastrin 1, 10 mM nicotinamide, 2.5 *μ*M thiazovivin, 10 *μ*M Y-27632, and 2.5 *μ*M CHIR99021 (Table 1).

### 2.8. EGFP Mouse-Derived Colon Organoid Transplantation into Recipient Mouse Colon

EGFP colon crypts were isolated from C57BL/6-Tg(CAG-EGFP) mouse, and EGFP colon organoids were grown for 5 days in both collagen and Matrigel matrix. Colon organoids were then isolated from collagen using collagenase from Clostridium histolyticum (Sigma) and Matrigel using ice-cold DPBS. EGFP colon organoids were suspended in a diluted media solution of Matrigel/70 *μ*l colon organoid culture media solution with 10 *μ*M Y-27632 at a ratio of 1 : 10. Prepared EGFP colon organoids were transplanted into the colonic lumen of EDTA colitis damaged C57BL/6 mouse using a 200 *μ*l pipette.

### 2.9. Quantitative Reverse Transcriptase-Polymerase Chain Reaction Analysis

The organoids (Passage 4) were treated with Cell Recovery Solution (Corning Incorporated, Corning, NY, USA) to remove the Matrigel, and total RNA was extracted using the MagListo™ 5 M Cell Total RNA Extraction Kit (Bioneer, Daejeon, Republic of Korea) according to the manufacturer's guidelines. Subsequently, the RNA product was subjected to a quantitative reverse transcription-polymerase chain reaction (RT-PCR). Firstly, RNA was synthesized to complementary DNA (cDNA) using AccuPower® RocketScript™ Cycle RT Premix (Bioneer). Quantitative polymerase chain reaction was performed by mixing the cDNA with AccuPower® 2X Greenstar qPCR Master Mix (Bioneer). The mRNA expression level was measured with Thermal Cycler Dice® Real Time System III (Takara, Shiga, Japan). The primer sequences are given in Table 2. The negative control (no cDNA template) was ran simultaneously with every assay, and PCR from each cDNA sample was ran in triplicate. Constitutively expressed GAPDH was used as an endogenous control to correct any potential variations in RNA loading or reaction efficiency. The results were presented as relative fold changes using the GAPDH reference and applying the formula 2^-ΔΔCt^. The PCR products were loaded into 1% agarose gel and visualized with a DNA gel staining solution under UV light.

### 2.10. WST-1 Activity Assay

Crypts isolated from the aforementioned organs were seeded into 96 multiwell plates and cultured. After 4 days of incubation, cell viability was assayed with a EZ-Cytox assay kit (Daeil Lab Service, Seoul, Republic of Korea) by adding water-soluble tetrazolium salt to each well followed by 3 more hours of incubation. After the 3-hour incubation, only the medium was transferred to new wells in another 96-well plate, and the absorbance was measured at a wavelength of 450 nm with a VersaMax ELISA Microplate Reader (Molecular Devices, CA, USA).

### 2.11. Immunofluorescence

Organoids immunocytochemistry had been developed previously [15]. Samples were thoroughly covered with primary antibodies at 4°C overnight. Antibodies used for the staining were as follows: mucin 2 (Santa Cruz Biotechnology, Santa Cruz, CA), Ki67 antibody (Abcam, Cambridge, MA), chromogranin A (Santa Cruz), lysozyme (Diagnostic BioSystems, Fremont, CA), mucin 5AC (Santa Cruz Biotechnology, Santa Cruz, CA), and villin (Santa Cruz Biotechnology, Santa Cruz, CA) The next day, the samples were embedded with secondary antibodies at room temperature for 2 h, and Alexa Fluor 594 conjugated anti-rabbit IgG and Alexa Fluor 594 conjugated anti-mouse IgG (Life Technologies, Gaithersburg, MD).

### 2.12. Data Analysis

Differences between 2 groups were assessed using Student's *t*-test. Furthermore, 3 or more groups were compared with one-way ANOVA followed by the Student-Newman-Keuls post hoc test. Differences were considered statistically significant at *p* < 0.05.

## 3. Results and Discussion

### 3.1. *In Vitro* Mouse Colon Organoids Culture in Collagen Matrix

**Figure 1:**
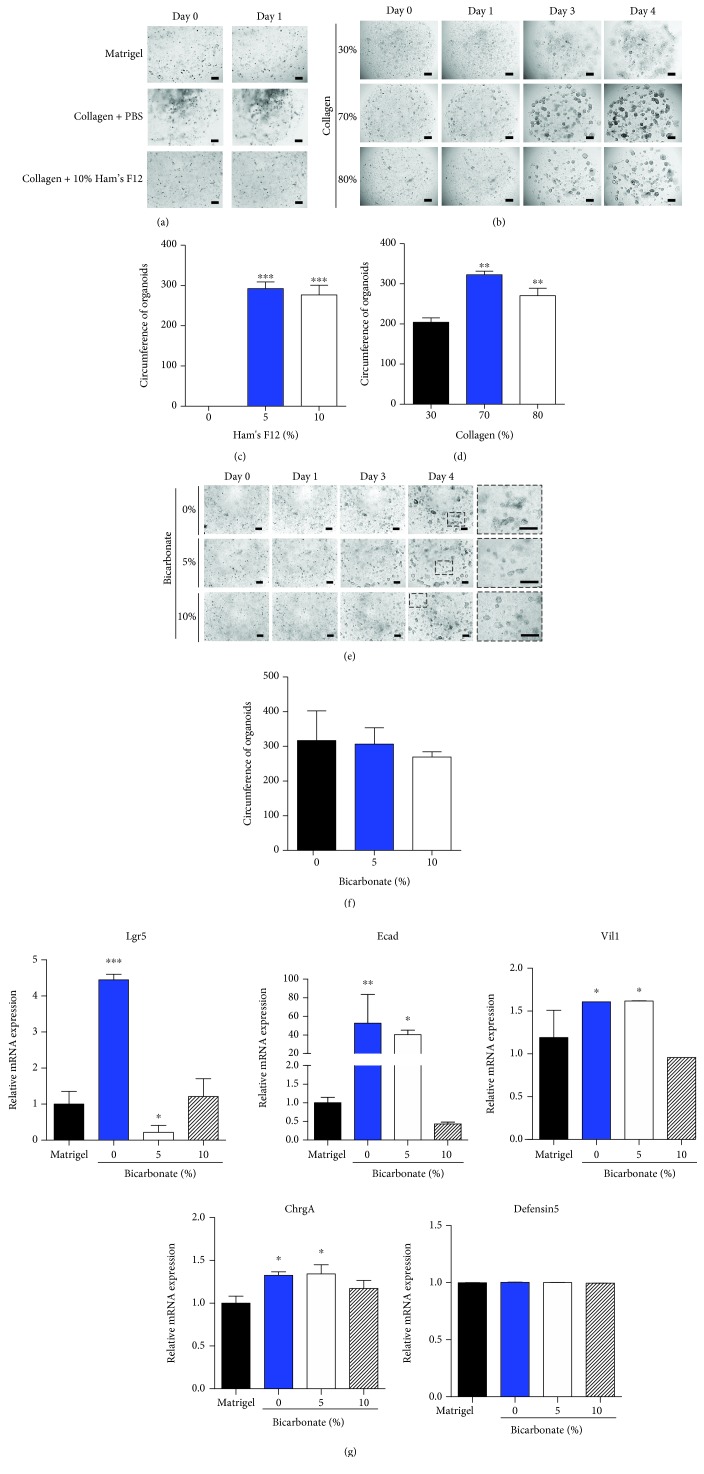
Effect of the components of collagen-based matrix on culturing mouse colon organoids. (a) Comparison of Matrigel (upper panel), collagen-based matrix without Ham's F12 (middle panel), and collagen-based matrix with Ham's F12 (down panel) shows different phenotypes. All matrices were mixed with the organoid at a ratio of 1 : 1. (b) Diverse percentages of collagen (30%, 70%, and 80%) distinguish different growth in each percentage. Organoids in Passage 0 were followed up on under a microscope daily. (c) Measured circumstance of the organoids shows the effect of the percentage of Ham's F12 (0%, 5%, and 10%). (d) Measured circumstance of the organoids with each collagen percentage rate (30%, 70%, and 80%). (e) Concentration of bicarbonate was tested and followed up daily under a microscope. (f) Measured circumstances of the organoids in different concentrations of bicarbonate were similar. (g) Relative mRNA expression in representative markers of the organoids was measured in Matrigel with 0%, 5%, and 10% bicarbonate. Lgr5 was used as a marker of intestinal stem cells, E-cadherin (Ecad) as a marker of epithelial cells, villin-1 (Vil1) as a marker of the epithelium border, defensin-5 (Defensin5) as a marker of Paneth cells, and chromogranin A (ChrgA) as a marker of enteroendocrine cells. The circumstance of the organoids was measured on the last day in vitro. Day denotes days of in vitro. Bar scale is 50 *μ*m. ^∗^*p* < 0.05, ^∗∗^*p* < 0.01, ^∗∗∗^*p* < 0.001.

The mouse colon crypts expanded and formed organ-like structures in vitro, both when grown in a collagen matrix and when grown in Matrigel. To determine the effect of Ham's F12, the ratio of Ham's F12 supplement was varied in the collagen matrix when culturing. The crypts were not able to grow as an organoid-like shape without Ham's F12, whereas in 10% of Ham's F12 complete colon organoid shape emerged, as occurred with the original ratio of collagen matrix (Figures 1(a) and 1(c)). The ratio of the collagen gel itself varied when the mouse colon crypts were cultured. The crypts were seeded in different percentages of collagen gel (30%, 70%, and 80%) and were cultured for the first 5 days at Passage 0. The results showed that at least 70% of collagen gel was required when culturing crypts in a 3D structure (Figures 1(b) and 1(d)). Unlike the previous two results, collagen-based 3D cultures grown with varying percentages of bicarbonate did not show significant differences in culturing organoids (Figures 1(e) and 1(f)). In order to determine the effect of the pH buffer in the collagen matrix, the cell lineage of the colon organoids in the culture systems based on Matrigel and on collagen with different percentages of bicarbonate was analyzed using a quantitative reverse-transcriptase polymerase chain reaction (RT-PCR). Organoids cultured in both Matrigel and collagen with 10% bicarbonate showed similar expression in stemness (Lgr5) and differentiated characteristics (E-cadherin, villin-1, chromogranin A, and defensin-5) of the colon organoids (Figure 1(g)). However, organoids in the collagen-based system without bicarbonate or with 5% bicarbonate showed quite different expression patterns of stemness and differentiation markers than those in the Matrigel-based system. This suggests that the bicarbonate ratio of collagen matrix components is quite important in culturing colon organoids.

**Figure 2:**
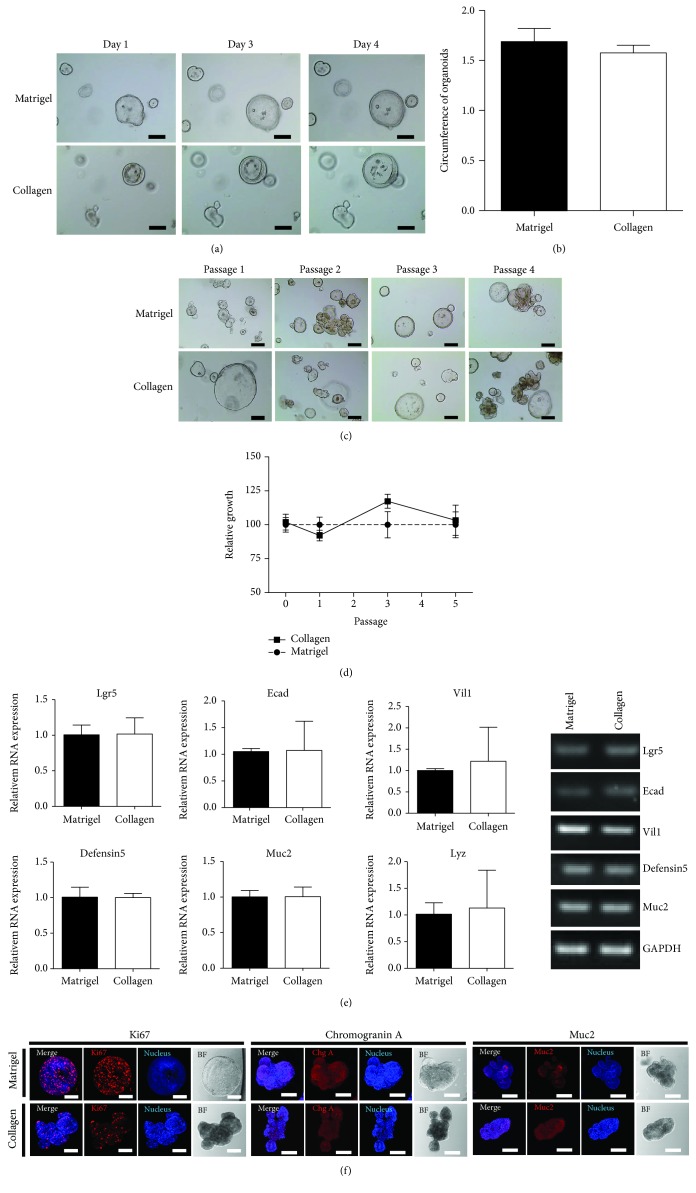
Maintenance of mouse colon organoids in Matrigel and in collagen-based matrix culture system. (a) Growth pattern of mouse colon organoid during Passage 3 in both Matrigel and collagen. Bar scale is 50 *μ*m. (b) Measured circumstance of mouse colon organoid in Passage 3. (c) In vitro growth of mouse colon organoids during Passage 5 in Matrigel and collagen matrix. (d) Organoid viability was measured using the WST assay. The organoids in Matrigel were used as a control. (e) Quantitative RT-PCR was performed to measure the expression of specific markers of colon organoids at Passage 4 cultured in both matrices (left). Muc2 (mucin 2) and Lyz (lysozyme) were used as markers of goblet cells and Paneth cells, respectively. Gene expression shown as gel electroporation (right). Day and Passage denote days of in vitro and passage number, respectively. Scale bar: 50 *μ*m. (f) The immunocytochemistry staining of Ki67, mucin 2, and chromogranin A was examined in Matrigel and Collagen matrix. Scale bar: 100 *μ*m.

The mouse colon organoid grown in the collagen matrix was composed of 80% collagen gel, 10% Ham's F12 supplement, and 10% bicarbonate and that grown in the Matrigel matrix maintained the crypts' growth. The mouse crypts cultured in these two matrices demonstrated normal morphology of the organoids (Figures 2(a) and 2(b)) and showed similar growth patterns during Passage 5 and above (Figures 2(c) and 2(d)). The quantitative RT-PCR demonstrated the expression of target genes in both culture methods (Figure 2(e)). Colon organoids in both the collagen- and Matrigel-based culture systems showed similar expression in stemness (Lgr5) and differentiated characteristics (E-cadherin, villin-1, defensin-5, mucin 2, and lysozyme) of the colon organoid (Figure 2(e)). The represent results of RT-PCR showed the same patterns as those of the quantitative RT-PCR. In addition, to identify the mouse colon organoid cell types, the cells were analyzed using immunocytochemistry staining with colon epithelium cell–specific markers. Cell markers Ki67, Muc2, and chromogranin A in colon epithelia were detected in both colon organoids grown in Matrigel and collagen matrix (Figure 2(f)), suggesting that colon organoids cultured in a collagen matrix may proliferate and serve its function as well as that of Matrigel.

### 3.2. *In Vitro* Mouse Small Intestinal Organoids Cultured in Collagen Matrix

**Figure 3:**
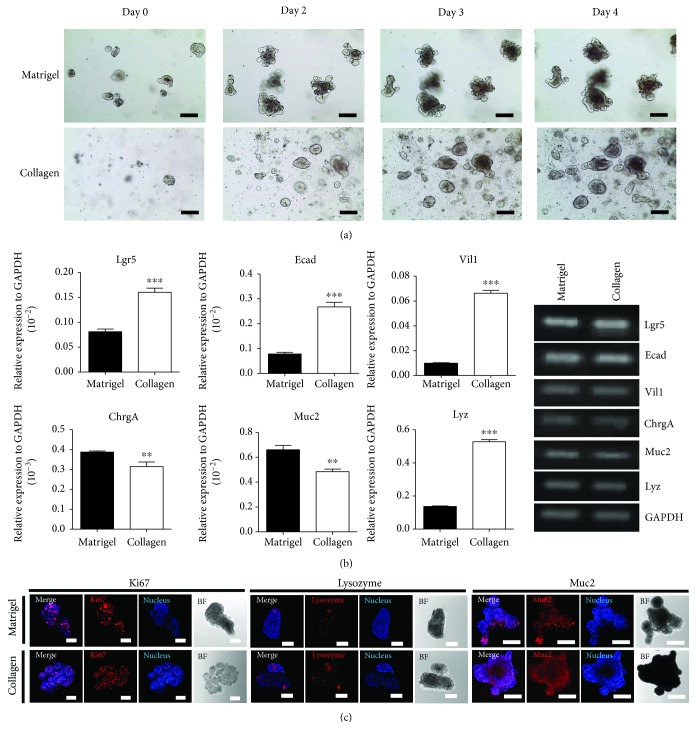
Comparison of growing mouse small intestinal organoids in Matrigel and in collagen-based matrix culture system. (a) Growth pattern and morphology of mouse small intestinal organoids in both Matrigel and collagen-based matrix during Passage 1. Bar scale is 50 *μ*m. (b) Quantitative RT-PCR analysis of the markers of intestinal organoids such as Lgr5, Ecad, Vil1, ChrgA, Muc2, and Lyz at Passage 4 cultured in both matrices (left). Gene expression shown as gel electroporation (right). ^∗∗^*p* < 0.01, ^∗∗∗^*p* < 0.001. (c) The immunocytochemistry staining of Ki67, mucin 2, and lysozyme was examined in Matrigel and Collagen matrix. Scale bar: 100 *μ*m.

It was found that small intestine organoid—may also be grown in both collagen- and Matrigel-based conditions. Crypts were isolated from the small intestine and cultured as mouse small intestinal organoids in both Matrigel- and collagen-based matrices. The small intestinal crypts in the collagen-based culture system appeared in their complete shape as organoids, as did those in the Matrigel-based culture system. Mouse small intestinal organoids showed a budded shape in Matrigel but not in collagen. Their morphologic changes in both collagen and Matrigel during Passage 4 and above were observed under a microscope as with the small intestinal organoids (Figure 3(a)), showing that organoids in the collagen matrix maintained their properties. To determine whether these two matrices affected their characteristics, a quantitative RT-PCR was performed which showed expressions of stemness (Lgr5) and differentiation markers (E-cadherin, villin-1, chromogranin A, mucin 2, and lysozyme) (Figure 3(b)). The expression level of the small intestine markers was significantly different between matrices, which may be due to the different rates of small intestine cellular composite in the culture systems. In particular, Lgr5, E-cadherin, villin-1, and lysozyme were expressed more in the small intestinal organoid cultured in collagen-based matrix than that of Matrigel. In addition, to identify the mouse small intestine organoid cell types, the cells were analyzed using immunocytochemistry staining with small intestinal epithelium cell–specific markers. Expression of cell markers such as Muc2, lysozyme, and Ki67 in small intestinal organoids cultured in collagen- and Matrigel-based matrices proved collagen-based matrix's compatibility and it serves to grow functioning organoids (Figure 3(c)).

### 3.3. *In Vitro* Mouse Stomach Organoid Cultured in Collagen Matrix

**Figure 4:**
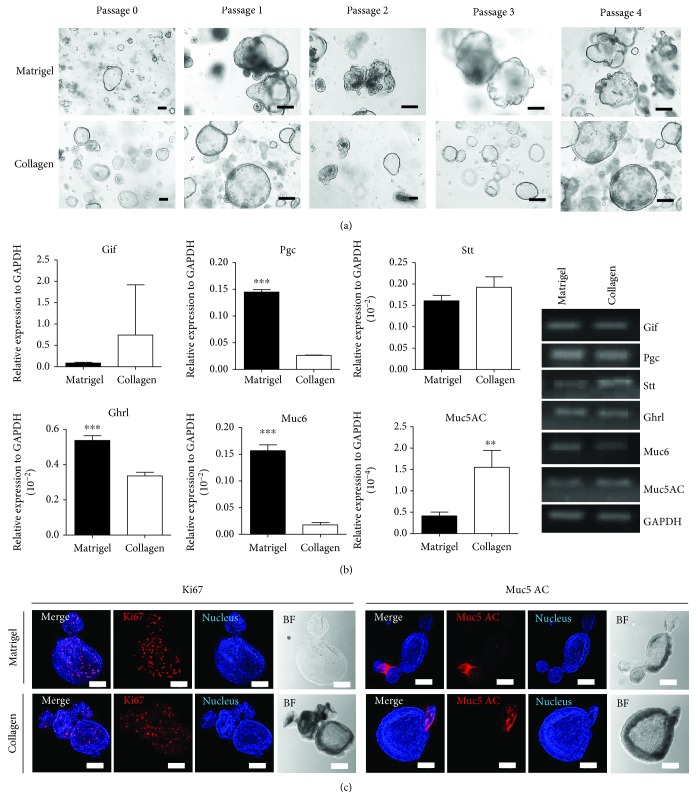
Comparison of growing mouse gland-derived stomach organoids in Matrigel and collagen-based matrix culture system. (a) Growth pattern and morphology of mouse gland–derived stomach organoids in both Matrigel and collagen-based matrix during Passages 0–4. Bar scale is 50 *μ*m. (b) Quantitative RT-PCR was performed to identify specific markers of stomach organoids at Passage 4 cultured in both matrices (left). Gastric intrinsic factor (gif) was used as a marker of parietal cells, pepsinogen (Pgc) as a marker of chief cells, somatostatin (Stt) as a marker of enteroendocrine cells, ghrelin (Ghrl) as a marker of ghrelin cells, mucin 6 (Muc6) as a marker of gland mucous cells, and mucin 5AC (Muc5AC) for a marker of gastric pit mucous cells. Gene expression shown as gel electroporation (right). ^∗∗^*p* < 0.01, ^∗∗∗^*p* < 0.001. (c) The immunocytochemistry staining of Ki67, mucin 5AC was examined in Matrigel and Collagen matrix. Scale bar: 100 *μ*m.

Gastric (stomach) glands from mice were isolated and cultured as stomach organoids in both Matrigel and collagen-based matrices. Initially, stomach crypts were scarce in the collagen-based matrix; they were found reliably on the third day of culture. The stomach crypts in the collagen-based culture system appeared in their complete shape as organoids, as were those in the Matrigel-based culture system (Figure 4(a)). Their morphology was observed daily by the microscope; the stomach organoids grew during Passage 4 or above without losing their characteristics. The quantitative RT-PCR revealed the expression of gastric-specific genes (Gif, Pgc, Stt, Ghrl, Muc6, and Muc5AC), showing different patterns in both matrices (Figure 4(b)). Particularly, Muc5AC were expressed more in stomach organoids grown in collagen matrix. In addition, to identify the mouse stomach organoid cell types, the cells were analyzed using immunocytochemistry staining with stomach epithelium cell–specific markers. Cell markers such as Ki67 and mucin 5AC were found in the gastric epithelia (Figure 4(c)).

### 3.4. *In Vitro* Human Colon Organoid Cultured in Collagen Matrix

**Figure 5:**
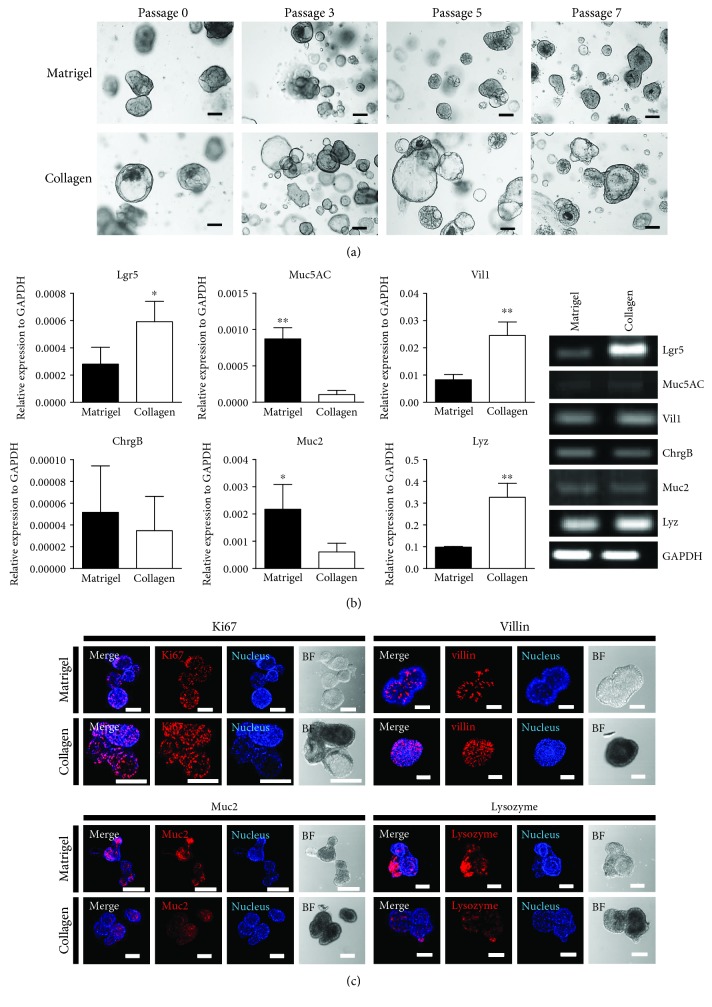
Comparison of growing human-derived colon organoids in Matrigel and in collagen-based matrix culture system. (a) Growth pattern and morphology of human large intestine organoids cultured in both Matrigel and collagen-based matrix during Passage 7. Bar scale is 50 *μ*m. (b) Quantitative RT-PCR was performed to identify specific markers (Lgr5, Muc5AC, Vil1, ChrgB, Muc2, and Lyz) of human large intestine at Passage 4 in both matrices (left). Chromogranin B (ChrgB) was used for targeting enteroendocrine cells. Gene expression shown as gel electroporation (right). ^∗^*p* < 0.05, ^∗∗^*p* < 0.01. (c) The immunocytochemistry staining of Ki67, villin, mucin 2, and lysozyme was examined in Matrigel and Collagen matrix. Scale bar: 100 *μ*m.

Human colon organoids were cultured in both matrices, as the mouse colon organoids could. The experiment was performed under the same conditions as those of the human organoid experiment, with the result that both matrices were found to provide an adequate culture system for growing human colon organoids. In both collagen and Matrigel conditions, human colon organoids maintained their characteristics up to Passage 7 (Figure 5(a)), and the quantitative RT-PCR results showed stemness (Lgr5) in both collagen and Matrigel but different expression patterns in the lineage markers (Figure 5(b)). Lgr5, villin-1, and lysozyme were expressed more in colon organoids cultured in collagen-based matrix than that of Matrigel. In addition, to identify the human colon cell types, the cells were analyzed using immunocytochemistry staining with human colon epithelium cell–specific markers. Cell markers such as Ki67, villin, Muc2, and lysozyme were found in the colon epithelia (Figure 5(c)).

### 3.5. *In Vivo* EGFP+ Transplantation of Mouse Colon Organoid Cultured in Collagen Matrix

**Figure 6:**
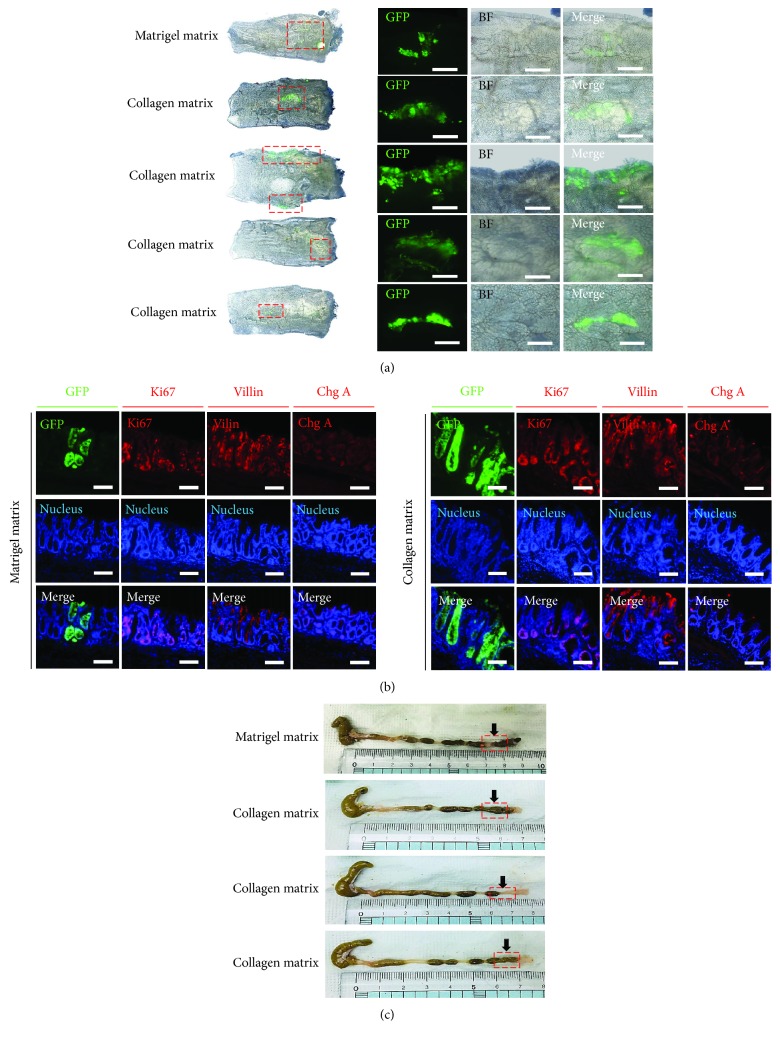
Transplantation of collagen matrix–cultured colon organoids into recipient mouse colonic epithelial. (a) C57BL/6 the recipient tissues were analyzed 7 days after transplantation. EGFP+ engraftments were present in both culture matrices (Matrigel and collagen). (b) Sections of the engrafted tissue 1 week posttransplantation show the presence of EGFP+ crypt cells, Ki67, villin, and chromogranin A differentiated cells in the crypts of donor origin, with Hoechst 33342 (nucleus) staining. Scale bar: 100 *μ*m. (c) Colonic lesion visual analysis of recipient mouse colon. Red box: engraftment site.

The mouse colon organoid engraftment model was derived from a mouse colon organoid in a collagen-based culture system. Colonic crypts were isolated from the CAG-EGFP mouse colon and cultured in a collagen matrix for 5 days. The EGFP colon organoid was transplanted into the colon of an EDTA injury mouse model as previously described [16]. One week after transplantation, an EGFP-expressive crypt was observed in the colonic epithelial tissue (Figures 6(a) and 6(b)). The EGFP+ crypts contained various differentiated cell types (Figure 6(b)). No lesions indicating rectal bleeding, prolapse, or diarrhea were observed (Figure 6(c)). These results indicate that collagen-based culture colon organoids have a possible clinical application for colon therapy.

## 4. Discussion

Maturing and maintaining healthy organoids require matrix to produce tissue-like environment, and several candidates have been investigated. First of all, Matrigel is well known to provide ECMs and growth factors and successfully support organoid growth, but its unknown elements have hindered clinical trials. For that reason, studies have been developed to find suitable substitute for Matrigel without tissue toxicity in vivo. Previously, alternative studies on hydrogel matrix were done [17, 18]. In these studies, hydrogel matrix successfully nurtured functioning organoids. However, hydrogel component also seemed to be toxic in vivo when transplanted. Unlike hydrogel, collagen is indeed the most abundant fibrous protein in ECM and constitutes up to 30% of its total protein mass, providing strength and regulating cellular mobility, chemotaxis, and development [19]. Characteristics of collagen matrix include simple structure, low immunogenicity, low cell-matrix interaction, and similar mechanical properties to that of in vivo. Therefore, mouse and human gastrointestinal tract organoids cultured in a collagen-based matrix may mimic original tissues more closely in morphology, gene expression, and molecular characteristics than those cultured in Matrigel [20–23].

The organ-specific miniature derived from stem cells and organ progenitors simulate in vivo system and overcome the limitations of spatial and cultural conditions [24]. Organ formation may generally be induced through the 3D culture of stem cells or progenitor cells. In this process, extracellular matrices secreted by the Engelbreth-Holm-Swarm tumor line and rich in laminin are used for 3D cultures. Through the optimization of various niche components such as Wnt, Noggin (BMP inhibitor), R-Spondin (Lgr5 receptor ligand), and epidermal growth factor (EGF) as well as niche growth factors, cells are successfully differentiated into functioning tissues. Mouse colon organoids cultured in a collagen-based matrix had similar morphogenesis to those cultured in Matrigel. Among our collagen-based cultures, different organoid morphology was observed in mouse small intestinal organoid compared to that of conventional Matrigel-based organoid. However, morphology did not have a major influence on the organoid's growth or function. Characteristics of specific messenger RNA (mRNA) markers were expressed in similar patterns in mouse colon organoids. Moreover, organoids cultured in collagen-based matrix had smaller circumference, smaller budding, and higher stability than those cultured in Matrigel, which formerly expected to maintain stemness better than the latter. With this findings, better fitness of transplanting collagen matrix–cultured colon organoids into in vivo intestinal environment was questioned, compared to that of Matrigel [16]. Successful transplantation outcome implemented superiority of collagen-based organoid culture in this context, but more detailed functional analysis like Forskolin-induced swelling test and colon organoids host–microbe interaction test should be done to offer quality reference as to prove safety and reliability of collagen-based organoid culture.

In this study, we showed that a collagen-based defined matrix for organoid may not only replace animal-derived undefined matrix for organoid culture but may also help to enhance the efficacy of organoids for developing therapeutics to repair epithelial wounds.

## 5. Conclusions

This study attempts to develop a new, collagen-based matrix that may serve as a substitute for Matrigel in the organoid culture method. This study suggests new culture methods of mouse colon organoids, small intestine–derived organoids, stomach-derived organoids, and human colon–derived organoids, all of which were successfully grown in the collagen-based matrix and had similar properties to those cultured in Matrigel.

## Figures and Tables

**Table 1:** Media component.

Reagent name	Company	Cat. No.	Mouse colon	Mouse small intestine	Mouse stomach	Human colon	Final concentration
Advanced DMEM/F12	Gibco	12634-028	O	O	O	O	1 X
Penicillin/streptomycin	Welgene Inc.	LS202-02	O	O	O	O	100 U/ml, *μ*g/ml
B-27 supplement	Gibco	17504-044	O	O	O	O	1 X
N-2 supplement	Gibco	17502-048	O	O	O	O	1 X
HEPES buffer solution	Gibco	15630-080	O	O	O	O	10 mM
GlutaMAX-I	Gibco	35050-061	O	O	O	O	2 mM
N-Acetylcysteine	Sigma-Aldrich	A7250	O	O	O	O	1.25 mM
Murine recombinant EGF	PeproTech	AF-315-09	O	O	O	O	50 ng/ml
Murine recombinant Noggin	PeproTech	250-38	O	O	O	O	100 ng/ml
R-Spondin-1 conditioned medium	Home-made		O	O	O	O	10%
Wnt3A conditioned medium	Home-made		O		O	O	50%
FGF10, 38-208aa human	ATGen	ATGP1387			O		200 ng/ml
A83-01	Tocris Bioscience	2939	O	O	O	O	500 nM
Nicotinamide	Sigma-Aldrich	N0636				O	10 mM
[Leu15]-Gastrin 1 human	Sigma-Aldrich	G9145			O	O	10 nM
Y-27632	STEMCELL Technologies	72304	O	O	O	O	10 *μ*M
CHIR99021	Sigma-Aldrich	SML1046	O	O	O	O	2.5 *μ*M
Thiazovivin	Sigma-Aldrich	SML1045				O	2.5 *μ*M
SB202190	Sigma-Aldrich	S7067				O	10 *μ*M

**Table 2:** PCR primer.

Species	Gene name	Direction	Sequence
Mouse	GAPDH	Forward	AACTTTGGCATTGTGGAAGG
		Reverse	ACACATTGGGGGTAGGAACA
Mouse	E-cadherin	Forward	ACCACTGCCCTCGTAATCGAA
		Reverse	CGTCCTGCCAATCCTGATGAA
Mouse	Lgr5	Forward	GACGCTGGGTTATTTCAAGTTCAA
		Reverse	CAGCCAGCTACCAAATAGGTGCTC
Mouse	Mucin-2	Forward	ATGCCCACCTCCTCAAAGAC
		Reverse	GTAGTTTCCGTTGGAACAGTGAA
Mouse	Lysozyme	Forward	GAGACCGAAGCACCGACTATG
		Reverse	CGGTTTTGACATTGTGTTCGC
Mouse	Defensin-5	Forward	AGGCTGATCCTATCCACAAAACAG
		Reverse	TGAAGAGCAGACCCTTCTTGGC
Mouse	Villin-1	Forward	GACGTTTTCACTGCCAATACCA
		Reverse	CCCAAGGCCCTAGTGAAGTCTT
Mouse	Chromogranin A	Forward	AAGGTGATGAAGTGCGTCCT
		Reverse	GGTGTCGCAGGATAGAGAGG
Human	GAPDH	Forward	GGTATCGTGGAAGGACTCATGAC
		Reverse	ATGCCAGTGAGCTTCCCGTTCAG
Human	Lgr5	Forward	TATGCCTTTGGAAACCTCTC
		Reverse	CACCATTCAGAGTCAGTGTT
Human	Mucin-5AC	Forward	TGATCATCCAGCAGCAGGGCT
		Reverse	CCGAGCTCAGAGGACATATGGG
Human	Mucin-2	Forward	CTGCACCAAGACCGTCCTCATG
		Reverse	GCAAGGACTGAACAAAGACTCAGAC
Human	Villin-1	Forward	GCAGCATTACCTGCTCTACGTT
		Reverse	GCTTGATAAGCTGATGCTGTAATTT
Human	Chromogranin B	Forward	CAGCCAACGCTGCTTCTC
		Reverse	TGGCATGGAATTGACAGC
Human	Lysozyme	Forward	CCGCTACTGGTGTAATGATGG
		Reverse	CATCAGCGATGTTATCTTGCAG
Mouse	Gastric intrinsic factor	Forward	TGAATCCTCGGCCTTCTATG
		Reverse	CAGTTAAAGTTGGTGGCACTTC
Mouse	Pepsinogen	Forward	CCAACCTGTGGGTGTCTTCT
		Reverse	TTAGGGACCTGGATGCTTTG
Mouse	Mucin 5AC	Forward	CCATGAAGTGGGAGTGTGTG
		Reverse	TTGGGATAGCATCCTTCCAG
Mouse	Mucin 6	Forward	TGCATGCTCAATGGTATGGT
		Reverse	TGTGGGCTCTGGAGAAGAGT
Mouse	Ghrelin	Forward	GCCCAGCAGAGAAAGGAATCCA
		Reverse	GCGCCTCTTTGACCTCTTCC
Mouse	Somatostatin	Forward	GAGGCAAGGAAGATGCTGTC
		Reverse	GGGCATCATTCTCTGTCTGG

## Data Availability

The data used to support the findings of this study are included within the article.

## References

[1] Ootani A., Li X., Sangiorgi E. (2009). Sustained in vitro intestinal epithelial culture within a Wnt-dependent stem cell niche. *Nature Medicine*.

[2] Sato T., Vries R. G., Snippert H. J. (2009). Single Lgr5 stem cells build crypt-villus structures in vitro without a mesenchymal niche. *Nature*.

[3] Bredenoord A. L., Clevers H., Knoblich J. A. (2017). Human tissues in a dish: the research and ethical implications of organoid technology. *Science*.

[4] Yui S., Nakamura T., Sato T. (2012). Functional engraftment of colon epithelium expanded in vitro from a single adult Lgr5^+^ stem cell. *Nature Medicine*.

[5] Fordham R. P., Yui S., Hannan N. R. F. (2013). Transplantation of expanded fetal intestinal progenitors contributes to colon regeneration after injury. *Cell Stem Cell*.

[6] Fukuda M., Mizutani T., Mochizuki W. (2014). Small intestinal stem cell identity is maintained with functional Paneth cells in heterotopically grafted epithelium onto the colon. *Genes & Development*.

[7] Nakamura T., Sato T. (2018). Advancing intestinal organoid technology toward regenerative medicine. *Cellular and Molecular Gastroenterology and Hepatology*.

[8] Anghelina M., Krishnan P., Moldovan L., Moldovan N. I. (2006). Monocytes/macrophages cooperate with progenitor cells during neovascularization and tissue repair. *The American Journal of Pathology*.

[9] Passaniti A., Taylor R. M., Pili R. (1992). A simple, quantitative method for assessing angiogenesis and antiangiogenic agents using reconstituted basement membrane, heparin, and fibroblast growth factor. *Laboratory Investigation*.

[10] Polykandriotis E., Arkudas A., Horch R. E., Kneser U., Mitchell G. (2008). To matrigel or not to matrigel. *American Journal of Pathology*.

[11] Tigges U., Hyer E. G., Scharf J., Stallcup W. B. (2008). FGF2-dependent neovascularization of subcutaneous Matrigel plugs is initiated by bone marrow-derived pericytes and macrophages. *Development*.

[12] Jabaji Z., Brinkley G. J., Khalil H. A. (2014). Type I collagen as an extracellular matrix for the in vitro growth of human small intestinal epithelium. *PLoS One*.

[13] Simian M., Bissell M. J. (2017). Organoids: a historical perspective of thinking in three dimensions. *Journal of Cell Biology*.

[14] Lee E.-S., Lim J.-Y., Im K.-I. (2015). Adoptive transfer of Treg cells combined with mesenchymal stem cells facilitates repopulation of endogenous Treg cells in a murine acute GVHD model. *PLoS One*.

[15] Hahn S., Nam M.-O., Noh J. H. (2017). Organoid-based epithelial to mesenchymal transition (OEMT) model: from an intestinal fibrosis perspective. *Scientific Reports*.

[16] Sugimoto S., Ohta Y., Fujii M. (2018). Reconstruction of the Human Colon Epithelium In~Vivo. *Cell Stem Cell*.

[17] Ono S., Ogawa R., Hyakusoku H. (2010). Complications after polyacrylamide hydrogel injection for soft-tissue augmentation. *Plastic and reconstructive surgery*.

[18] Gjorevski N., Sachs N., Manfrin A. (2016). Designer matrices for intestinal stem cell and organoid culture. *Nature*.

[19] Frantz C., Stewart K. M., Weaver V. M. (2010). The extracellular matrix at a glance. *Journal of Cell Science*.

[20] Jabaji Z., Sears C. M., Brinkley G. J. (2013). Use of collagen gel as an alternative extracellular matrix for the in vitro and in vivo growth of murine small intestinal epithelium. *Tissue Engineering Part C Methods*.

[21] Whitehead R. H., Brown A., Bhathal P. S. (1987). A method for the isolation and culture of human colonic crypts in collagen gels. *In Vitro Cellular & Developmental Biology*.

[22] Wildrick D. M., Roll R., Lointier P., Quintanilla B., Nichols D. H., Boman B. M. (1997). Isolation of normal human colonic mucosa: comparison of methods. *In Vitro Cellular & Developmental Biology – Animal*.

[23] Sachs N., Tsukamoto Y., Kujala P., Peters P. J., Clevers H. (2017). Intestinal epithelial organoids fuse to form self-organizing tubes in floating collagen gels. *Development*.

[24] Lancaster M. A., Knoblich J. A. (2014). Organogenesis in a dish: modeling development and disease using organoid technologies. *Science*.

